# Association of ADH1B polymorphism and alcohol consumption with increased risk of intracerebral hemorrhagic stroke

**DOI:** 10.1186/s12967-021-02904-4

**Published:** 2021-05-29

**Authors:** Chun-Hsiang Lin, Oswald Ndi Nfor, Chien-Chang Ho, Shu-Yi Hsu, Disline Manli Tantoh, Yi-Chia Liaw, Mochly-Rosen Daria, Che-Hong Chen, Yung-Po Liaw

**Affiliations:** 1 Department of Public Health and Institute of Public Health, Chung Shan Medical University, No. 110 Sec. 1 Jianguo N. Road, Taichung, 40201 Taiwan; 2 Department of Neurology, Yuanlin Christian Hospital, Yuanlin, Changhua County 510 Taiwan; 3 Department of Chemical and Systems Biology, Stanford University School of Medicine, Stanford, CA 94305 USA; 4 Department of Physical Education, Fu Jen Catholic University, New Taipei City, 24205 Taiwan; 5 Research and Development Center for Physical Education, Health and Information Technology, Fu Jen Catholic University, New Taipei City, 24205 Taiwan; 6 Department of Medical Education, Taipei Veterans General Hospital, Taipei, 11217 Taiwan; 7 Department of Medical Imaging, Chung Shan Medical University Hospital, Taichung City, 40201 Taiwan

**Keywords:** Cardiovascular disease, Stroke, Epidemiology

## Abstract

**Background:**

Alcohol consumption is one of the modifiable risk factors for intracerebral hemorrhage, which accounts for approximately 10–20% of all strokes worldwide. We evaluated the association of stroke with genetic polymorphisms in the alcohol metabolizing genes, alcohol dehydrogenase 1B (ADH1B, rs1229984) and aldehyde dehydrogenase 2 (ALDH2, rs671) genes based on alcohol consumption.

**Methods:**

Data were available for 19,500 Taiwan Biobank (TWB) participants. We used logistic regression models to test for associations between genetic variants and stroke. Overall, there were 890 individuals with ischemic stroke, 70 with hemorrhagic stroke, and 16,837 control individuals. Participants with ischemic but not hemorrhagic stroke were older than their control individuals (mean  ±  SE, 58.47 ± 8.17 vs. 48.33 ± 10.90 years, p  <  0.0001). ALDH2 rs671 was not associated with either hemorrhagic or ischemic stroke among alcohol drinkers. However, the risk of developing hemorrhagic stroke was significantly higher among ADH1B rs1229984 TC  +  CC individuals who drank alcohol (odds ratio (OR), 4.85; 95% confidence interval (CI) 1.92–12.21). We found that the test for interaction was significant for alcohol exposure and rs1229984 genotypes (p for interaction  =  0.016). Stratification by alcohol exposure and ADH1B rs1229984 genotypes showed that the risk of developing hemorrhagic stroke remained significantly higher among alcohol drinkers with TC  +  CC genotype relative to those with the TT genotype (OR, 4.43, 95% CI 1.19–16.52).

**Conclusions:**

Our study suggests that the ADH1B rs1229984 TC  +  CC genotype and alcohol exposure of at least 150 ml/week may increase the risk of developing hemorrhagic stroke among Taiwanese adults.

**Supplementary Information:**

The online version contains supplementary material available at 10.1186/s12967-021-02904-4.

## Background

The relationship between consumption of alcoholic drinks and stroke (including hemorrhagic and ischemic stroke) has been a long-standing debate, and results from previous epidemiological studies have been inconclusive [1]. Alcohol may contribute to stroke by (1) inducing cardiac arrhythmias, which causes cardiogenic embolic stroke, [2] (2) increasing the risk of hypertension in a linear positive dose-response manner in men and a J-shaped dose-response fashion in women [3]. In their meta-analysis to summarize the evidence from prospective studies on alcohol drinking and stroke types, Larsson et al. [1] suggested that light and moderate alcohol consumption was inversely associated with ischemic stroke: in contrast, heavy drinking was associated with increased risk of all stroke with a stronger association for hemorrhagic strokes.

Alcohol metabolism is one of the biological determinants that can significantly influence drinking behavior and alcohol-related organ damage [4]. Two key enzymes, alcohol dehydrogenase 1B (ADH1B) and aldehyde dehydrogenase 2 (ALDH2) are required to convert from alcohol to acetaldehyde and, eventually to acetic acid. Genetic polymorphisms may increase the risk of alcohol addiction. Two single-nucleotide polymorphisms (SNP), rs1229984 (ADH1B), and rs671 (ALDH2), which are highly prevalent in Asians, have been shown to encode different versions of ADH and ALDH [5]. The fast alcohol metabolizing ADH1B variant (ADH1B rs1229984 T or *2 allele vs. C or *1 allele), and the inactive ALDH2 variant (ALDH2 rs671 A or *2 allele vs. G or *1 allele) cause rapid acetaldehyde accumulation and unpleasant alcohol flushing reaction; hence may inhibit alcohol consumption. The ADH1B T allele encodes a superactive enzyme subunit that has about 40 times faster maximum velocity than the enzyme encoded by the ADH1B C allele [6], and carriers of the ALDH2 inactive A allele has only about 0–17% of the residual activity as compared to the non-carriers [7]. These two genetic variants are more prevalent among East Asians (~ 90% ADH1B T allele carriers and  ~ 40% ALH2 A allele carriers, respectively) than Caucasians (~ 10% ALDH1B T allele carriers and  ~ 0% ALDH2 A allele carriers, respectively) [8, 9]. Even among different Asian ethnic groups, the prevalence of these two genetic polymorphisms varies. For example, the ALDH2*2 allele appears to be most prevalent in Chinese–American, Han Chinese, and Taiwanese, Japanese, and Korean samples. Much lower rates have been reported among Thais, Filipinos, Indians, and Chinese and Taiwanese aborigines [10].

Associations between stroke and these alcohol metabolism-related variants have not been widely reported in Asia. A meta-analysis of 83 prospective studies with 5,99,912 current alcoholic beverage drinkers mainly of European descent suggested that stroke incidence increased steadily with the alcohol intake [11]. This aligns with epidemiologic and genetic studies in China where U-shaped associations were found between self-reported alcohol intake and the incidence of ischemic stroke, intracerebral hemorrhage, and total stroke [12, 13]. Moderate alcohol intake (100 g per week) was associated with a lower risk of stroke [13]. However, genetic analyses showed no associations with ischemic stroke, intracerebral hemorrhage, or total stroke. We examined the relationship between genetic polymorphisms of ADH1B rs1229984, ALDH2 rs671, and stroke in relation to alcohol consumption among Taiwanese adults.

## Methods

### Data source

Data used in this study were obtained from two data resources: (1) The National Health Insurance Research Database (NHIRD), which had medical records for patients diagnosed with specific disease (ischemic stroke, hemorrhagic stroke, diabetes, hypertension, hypertension, and atrial fibrillation) between 1998 and 2015 and (2) TWB, an established national health resource that contained basic demographic, physical assessment, biochemistry, questionnaire, and genotype data collected from 2008 to 2015. These databases were linked at the Health and Welfare Data Science Center (HWDC) using personal identification numbers (PIN).

### Patient identification

Our initial recruitment included 19,500 TWB participants. We retrieved information on their demographic (sex, age, body mass index [BMI]), lifestyle (regular exercise, smoking, and alcohol consumption), and genotype data from the TWB Database. We excluded persons with; incomplete questionnaire and genotype information (n  =  1636), both ischemic and hemorrhagic stroke (n  =  40), alcoholic hepatitis (n  =  16), and those who started to drink after developing stroke (n  =  11), leaving specifically those with ischemic stroke (n  =  890), hemorrhagic stroke (n  =  70), and 16,837 control individuals in the final analysis. Alcohol drinkers were defined as persons who reported drinking more than 150 ml of alcohol per week during the 6 months before health examination. Physical activity was defined as any amount of exercise activity at least three times a week and lasting for at least 30 min each time. Current smokers were defined as persons who had been smoking for the past 6 months, and continued to smoke during assessment. Never smokers were those who have never smoked or have been smoking for less than 6 months. Former smokers were persons who had smoked before but later decided to quit. A secondhand smoker was defined as a person who was currently exposed to cigarette smoke from other people for at least 5 min per day.

### Genetic variant selection

We searched peered-reviewed literature databases to select the HLA polymorphic variants (ALDH2 rs671 and ADH1B rs1229984) previously associated with alcohol metabolism. TWB genotyping was performed using the custom Taiwan Biobank chips. The Axiom™ Genome-Wide ASI Array (Affymetrix, Santa Clara, CA, USA) was used for genotyping. For the TWB SNPs array, we followed a standard quality control procedure to exclude SNP with a low call rate (< 95%), a p value for the Hardy–Weinberg equilibrium test of  < 1.0 × 10^−3^, and minor allele frequency of  < 0.05. Multivariate unconditional logistic regression analysis was used to estimate the odds ratios with their 95% confidence intervals.

### Definition of outcomes

Patients were defined as having ischemic or hemorrhagic stroke if they had either two outpatient visits or one-time hospitalization in NHIRD from 1998 to 2015 with reported International Classification of Diseases, Ninth Revision, Clinical Modification (ICD-9 CM) 433–437 and 430–432, respectively. The potential confounders included co-morbidities (hypertension (ICD-9-CM: 401–405), diabetes mellitus (ICD-9-CM: 250), hyperlipidemia (ICD-9-CM: 272), and atrial fibrillation (ICD-9-CM: 427.3). The Institutional Review Board of Chung Shan Medical University approved this study.

### Statistical analysis

We used the statistical analysis system (SAS) software (version 9.4) and PLINK 1.09 to perform statistical analyses. We used the chi-square test and t test for discrete and continuous variables. We also used Fisher’s exact test to determine associations between categorical variables. Logistic regression analysis was used to investigate the effects of alcohol drinking on ischemic stroke and hemorrhagic stroke. We estimated the odds ratios (ORs) with their 95% confidence intervals.

## Results

Data linkage was established between TWB and the NHIRD for 19,500 participants. Overall, reliable data of 890 individuals with ischemic stroke, 70 with hemorrhagic stroke, and 16,837 controls were available for analysis (Table 1). Participants with ischemic but not hemorrhagic stroke, were older (mean  ±  SE, ischemic stroke, 58.47 ± 8.17 years vs. control group, 48.33 ± 10.90 years, p < 0.0001; hemorrhagic stroke, 50.54 ± 10.60 years vs. control group, 48.33 ± 10.90 years, p = 0.0897) and had a lower education status. The prevalence of cerebrovascular disease risk factors such as hypertension, hyperlipidemia, alcohol intake, and diabetes was significantly different among patients with both ischemic and hemorrhagic stroke compared with the control individuals (p  <  0.05).

**Table 1:** Descriptive data of the participants

	Control n = 16,837		Ischemic stroke n = 890			Hemorrhagic stroke n = 70		
	n	%	n	%	*p *value	n	%	*p *value
ALDH2 rs671					0.6642			0.8104
GG	8554	50.80	458	51.46		33	47.14	
GA	6832	40.58	363	40.79		31	44.29	
AA	1451	8.62	69	7.75		6	8.57	
ADH1B rs1229984					0.3012			0.7299
TT	9159	54.40	477	53.60		39	55.71	
TC	6568	39.01	364	40.90		25	35.71	
CC	1110	6.59	49	5.51		6	8.57	
Sex					0.0874			0.2084
Women	8724	51.81	435	48.88		31	44.29	
Men	8113	48.19	455	51.12		39	55.71	
Age (mean ± SD)	**48.33 ± 10.90**		**58.47 ± 8.17**		**< 0.0001**	50.54 ± 10.60		0.0897
Educational level					**< 0.0001**			0.1665
Elementary school	902	5.36	115	12.92		6	8.57	
Junior/senior high school	6498	38.59	418	46.97		32	45.71	
University and above	9437	56.05	357	40.11		32	45.71	
Smoking (ref: never)					**0.0051**			0.2571
Nonsmoker	12,732	75.62	664	74.61		47	67.14	
Former smoker	2124	12.62	141	15.84		12	17.14	
Current smoker	1981	11.77	85	9.55		11	15.71	
Alcohol intake					0.0819			**0.0212**
No	15,119	89.80	783	87.98		57	81.43	
Yes	1718	10.20	107	12.02		13	18.57	
Physical activity					**< 0.0001**			0.9507
No	10,041	59.64	376	42.25		42	60.00	
Yes	6796	40.36	514	57.75		28	40.00	
BMI, kg/m^2^ (mean ± SD)	24.23 ± 3.63		24.82 ± 3.65		**< 0.0001**	24.64 ± 4.08		0.3547
Diabetes					**< 0.0001**			**0.0204**
No	14,767	87.71	569	63.93		55	78.57	
Yes	2070	12.29	321	36.07		15	21.43	
Hypertension					**< 0.0001**			**0.0189**
No	13,246	78.67	352	39.55		47	67.14	
Yes	3591	21.33	538	60.45		23	32.86	
Hyperlipidemia					**< 0.0001**			**0.0148**
No	12,076	71.72	326	36.63		41	58.57	
Yes	4761	28.28	564	63.37		29	41.43	

*SD* standard deviation; *BMI* body mass index

Bold font indicates statistical significance from publication

We found that the ADH1B rs1229984 variant was not associated with ischemic stroke based on alcohol intake (Table 2). However, we found significant associations with diabetes, hypertension, and hyperlipidemia among patients with rs1229984 TT and TC  +  CC genotypes. Among TT individuals, the OR (95% CI) was 1.43 (1.15–1.79), 2.58 (2.06–3.24), and 1.48 (1.18–1.85) for those with diabetes, hypertension, and hyperlipidemia, respectively. Similarly, the OR estimates among TC  +  CC individuals with these conditions were 1.53 (1.20–1.94), 2.04 (1.61–2.60), and 1.79 (1.40–2.28), respectively. But, importantly, the risk of hemorrhagic stroke was higher among carriers of the ADH1B, rs1229984 TC  +  CC (heterozygous or homozygous genotype), who drank alcohol (OR, 4.85; 95% CI 1.92–12.21, p  =  0.0008) as shown in Table 3. This elevated risk of hemorrhagic stroke with alcohol consumption was only observed among carriers with at least one slow alcohol metabolizing allele of ADH i.e., rs1229984 TC  +  CC genotypes) and not among those with the TT homozygous genotype. The test for interaction was significant for alcohol intake and rs1229984 genotypes (p for interaction  =  0.016). After stratification by alcohol intake (Table 4), the odds remained significantly higher among the slow ADH1B rs1229984 TC or CC individuals who consumed alcohol (OR  =  4.43, 95% CI 1.19–16.52, p  =  0.0266). Among non-alcohol drinkers, no significant association was found between the rs1229984 genotype and hemorrhagic stroke risk. We also found that the ALDH2 inactive enzyme rs671 polymorphism was not associated with either ischemic stroke or hemorrhagic stroke among alcohol drinkers. The corresponding ORs for hemorrhagic and ischemic stroke among GA  +  AA compared to GG alcohol drinkers were 0.65 (95% CI 0.17–2.43, p  =  0.5226; Table 4), and 1.26 (95% CI 0.80–1.98, data not shown), respectively.

**Table 2:** Association of ischemic stroke with alcohol intake by rs1229984

	rs1229984 (TT)			rs1229984 (TC/CC)		
	OR	95% CI	*p* value	OR	95% CI	*p* value
Alcohol intake (ref: no)						
Yes	1.16	0.83–1.62	0.3954	0.99	0.70–1.39	0.9334
rs671 (ref: GG)						
GA/AA	1.14	0.93–1.38	0.2065	0.84	0.68–1.03	0.0933
Sex (ref: women)						
Men	0.91	0.72–1.15	0.4151	1.20	0.93–1.54	0.1577
Age	1.08	1.06–1.09	**< 0.0001**	1.07	1.05–1.08	**< 0.0001**
Educational level (ref: elementary school)						
Junior/senior high school	1.18	0.86–1.62	0.3039	1.04	0.74–1.46	0.8190
University and above	0.98	0.70–1.37	0.9192	0.83	0.58–1.19	0.3111
Smoking (ref: nonsmoker)						
Former smoker	0.78	0.57–1.07	0.1198	1.06	0.78–1.46	0.6970
Current smoker	1.07	0.75–1.53	0.7148	0.86	0.58–1.27	0.4455
Physical activity (ref: no)						
Yes	0.97	0.79–1.19	0.7897	1.12	0.90–1.39	0.3182
BMI	1.00	0.97–1.03	0.9358	1.00	0.97–1.03	0.9962
Diabetes (ref: no)						
Yes	1.43	1.15–1.79	**0.0016**	1.53	1.20–1.94	**0.0006**
Hypertension (ref: no)						
Yes	2.58	2.06–3.24	**< 0.0001**	2.04	1.61–2.60	**< 0.0001**
Hyperlipidemia (ref: no)						
Yes	1.48	1.18–1.85	**0.0008**	1.79	1.40–2.28	**< 0.0001**

*OR* odds ratio; *CI* 95% confidence interval; *BMI* body mass index

Bold font indicates statistical significance from publication

**Table 3:** Association of hemorrhagic stroke with alcohol intake by rs1229984

	ADH1B rs1229984 (TT)			ADH1B rs1229984 (TC/CC)		
	OR	95% CI	*p* value	OR	95% CI	*p* value
Alcohol intake (ref: no)						
Yes	0.52	0.15–1.83	0.3119	**4.85**	**1.92–12.21**	**0.0008**
ALDH2 rs671 (ref: GG)						
GA/AA	0.82	0.43–1.56	0.5510	2.00	0.95–4.20	0.0683
Sex (ref: women)						
Men	1.55	0.73–3.31	0.2577	0.77	0.31–1.89	0.5679
Age	1.02	0.98–1.05	0.4322	0.99	0.95–1.03	0.6938
Educational level (ref: elementary school)						
Junior/senior high school	0.70	0.22–2.19	0.5359	1.12	0.24–5.16	0.8886
University and above	0.59	0.18–1.96	0.3923	0.75	0.15–3.75	0.7239
Smoking (ref: nonsmoker)						
Former smoker	1.38	0.57–3.36	0.4792	0.86	0.25–2.91	0.8042
Current smoker	0.88	0.28–2.75	0.8199	1.38	0.48–3.99	0.5488
Physical activity (ref: no)						
Yes	0.71	0.36–1.43	0.3407	1.00	0.46–2.15	0.9931
BMI	1.03	0.94–1.13	0.5331	0.95	0.85–1.06	0.3365
Diabetes (ref: no)						
Yes	1.36	0.58–3.18	0.4818	1.44	0.51–4.06	0.4881
Hypertension (ref: no)						
Yes	0.93	0.41–2.09	0.8536	1.93	0.78–4.77	0.1558
Hyperlipidemia (ref: no)						
Yes	1.63	0.76–3.49	0.2072	1.08	0.44–2.67	0.8733

*OR* odds ratio; *CI* 95% confidence interval; *BMI* body mass index

Bold font indicates statistical significance from publication

**Table 4:** Association of hemorrhagic stroke with rs1229984 based on alcohol intake

	No alcohol intake			Alcohol intake		
	OR	95% CI	*p* value	OR	95% CI	*p *value
rs671_A (ref: GG)						
GA/AA	1.34	0.79–2.28	0.2808	0.65	0.17–2.43	0.5226
rs1229984_C (ref: TT)						
TC/CC	0.70	0.41–1.21	0.2000	**4.43**	**1.19–16.52**	**0.0266**
Sex (ref: women)						
Men	1.30	0.71–2.40	0.3988	0.44	0.12–1.70	0.2346
Age	1.02	0.98–1.05	0.3446	0.95	0.89–1.02	0.1748
Educational level (ref: elementary School)						
Junior/senior high school	1.14	0.38–3.38	0.8172	0.21	0.04–1.28	0.0899
University and above	0.91	0.30–2.83	0.8752	0.11	0.01–0.84	0.0333
Smoking (ref: nonsmoker)						
Former smoker	1.26	0.57–2.78	0.5705	0.81	0.17–3.76	0.7854
Current smoker	1.08	0.43–2.71	0.8666	1.04	0.26–4.13	0.9508
Physical activity (ref: no)						
Yes	0.70	0.39–1.25	0.2294	2.01	0.60–6.71	0.2545
BMI	1.01	0.94–1.09	0.7923	0.92	0.77–1.09	0.3157
Diabetes (ref: no)						
Yes	1.16	0.55–2.45	0.6919	3.52	0.79–15.66	0.0991
Hypertension (ref: no)						
Yes	1.22	0.63–2.39	0.5589	1.57	0.38–6.50	0.5328
Hyperlipidemia (ref: no)						
Yes	1.47	0.78–2.78	0.2333	0.86	0.20–3.75	0.8361

*OR* odds ratio; *CI* 95% confidence interval; *BMI* body mass index

Bold font indicates statistical significance from publication

## Discussion

In this population-based study, we were able to link genetic, clinical, and lifestyle data from TWB Database to medical records from the NHIRD using PINs for 19,500 residents in Taiwan; these data enabled more detailed and accurate analyses at the individual level. We examined the relationship among stroke, alcohol consumption, potential confounding factors, and SNPs rs1229984 and rs671 in two important alcohol metabolizing genes, ADH1B and ALDH2, respectively. Our main finding suggested that ADH1B rs1229984 TC  +  CC genotype was a substantial risk factor (OR  >  4.0) for hemorrhagic stroke in patients who drank more than 150 ml of alcohol per week. A large prospective randomized study in China indicated that genotype-predicted mean alcohol intake had a continuously positive log-linear association with stroke risk, which was stronger for intracerebral hemorrhage (relative risk per 280 g/week: 1.58, 95% CI 1.36–1.84) than for ischemic stroke (1.27, 95% CI 1.13–1.43) [13]. However, the study did not distinguish the specific effects of ADH1B and ALDH2 genotypes on hemorrhagic stroke in alcohol-drinking patients. Our study revealed that the ADH1B rs1229984 TC  +  CC genotype, but not the slow acetaldehyde metabolizing ALDH2 rs671 variant increased the risk of hemorrhagic stroke with alcohol exposure. The underlying mechanism for our observed association is not completely clear. Alcohol drinkers with the ADH1B TC  +  CC genotype may be predisposed to hemorrhagic stroke risk irrespective of the amount of alcohol intake. Since the slower alcohol metabolizing ADH1B TC/CC genotype is known to increase susceptibility to alcoholism [14–16] in a culture that encourages drinking after work-related functions, [14–16] a heavier intake may lead to high blood pressure, reduced platelet aggregation, and enhanced fibrinolysis, which may elevate the risk of hemorrhagic events [17]. Unfortunately, TWB data did not contain detailed information on the quantity and frequency of alcohol consumption. We distinguished alcohol drinkers from non-drinkers based on a weekly consumption of at least 150 ml. Therefore, we could not assess the hemorrhagic stroke risk based on the absolute amount of alcohol consumed.

The rs671 A and rs1229984 T alleles are common in Asia populations [18]. In contrast, the rs671 A allele is almost absent among Caucasians: the rs1229984 C allele is the dominant allele among Caucasians but is less common among Asians (about 5–7%) [18]. Although previous studies have evaluated the interaction between alcohol drinking and ADH1B and ALDH2 variants, they mainly focused on ischemic stroke and ALDH2 rs671. In one study, rs671 appeared to be an independent risk factor for ischemic stroke among Taiwanese men [19, 20]. In another study, no association was found between rs1229984 TT and stroke among the Han Chinese [21].

The major limitation of our study is that information on alcohol exposure was based on self-report. This was more likely to introduce recall bias, which may have led to patient misclassification. In addition, we did not have information on alcohol type and the absolute amount of daily consumption in the questionnaires used. Also, we had a small sample size of hemorrhagic stroke patients who drank alcohol (n  =  13). Among these individuals, those with TT, TC, and CC alleles for rs1229984 were 3, 9, 1, respectively (Additional file 1: Table S1). Of note, validation of our findings in a second cohort would have improved this study’s strength. However, this was not possible since such data are currently not available in our data sources. Next, the control cases had significantly fewer patients with DM, hypertension, and hyperlipidemia than stroke patients. Despite these, we adjusted for these covariates in our regression models. Finally, no clinical laboratory data were recorded in the NHIRD. Therefore, we could not determine patients with alcohol abuse based on aberrations in liver enzymes and platelets. Nevertheless, in our analyses, we identified a correlation between specific alcohol-related genetic variations and the risk of hemorrhagic stroke in Taiwan. Future studies should examine hemorrhagic stroke risk and its association with ADH1B rs1229984 polymorphism and alcohol consumption in other ethnic groups, including East Asians and Caucasians.

## Conclusions

Our study suggests that ADH1B rs1229984 TC  +  CC genotype, resulting in a slow ethanol conversion to acetaldehyde and alcohol exposure of at least 150 ml/week may increase hemorrhagic stroke risk among Taiwanese adults. A real-world randomized control study including gene-environment interactions in diverse populations is crucial in understanding the underlying mechanisms of this potential association between stroke, alcohol use, and genetic susceptibility.

## Supplementary Information

**Additional file 1: Table S1.** Study participants based on alcohol exposure and rs671 and rs1229984 variant genotypes.

## Data Availability

The data that support the findings of this study are available from the Taiwan Biobank data source but restrictions apply to the availability of these data, which were used under license for the current study, and so are not publicly available. Data are however available from the authors upon reasonable request and with permission of Taiwan Biobank.

## Abbreviations

ICD-9-CM: International Classification of Diseases Clinical Modification, 9th revision
BMI: Body mass index
OR: Odds ratio
CI: 95% Confidence interval
TWB: Taiwan biobank
NHIRD: National Health Insurance Research Database
ALDH2: Aldehyde dehydrogenase 2
