# Supplementary material for: Association of ADH1B polymorphism and alcohol consumption with increased risk of intracerebral hemorrhagic stroke
Source: J Transl Med. 2021 May 29;19:227. doi: 10.1186/s12967-021-02904-4 (PMC8164791; doi:10.1186/s12967-021-02904-4)
Supplement: Supplementary file 1 — Additional file 1: Table S1. Study participants based on alcohol exposure and rs671 and rs1229984 variant genotypes. [file 12967_2021_2904_MOESM1_ESM.docx]

Supplementary Table 1. Study participants based on alcohol exposure and rs671 and rs1229984 variant genotypes.

|  | **No alcohol intake** | |  | **Alcohol intake** | |  | **P-value** |
| --- | --- | --- | --- | --- | --- | --- | --- |
|  | **N** | **%** |  | **N** | **%** |  | **Fisher test** |
| ***Controls*** | | | | | | | |
| **rs671** |  |  |  |  |  |  | <.0001 |
| **GG** | 7332 | 48.50 |  | 1222 | 71.13 |  |  |
| **GA** | 6355 | 42.03 |  | 477 | 27.76 |  |  |
| **AA** | 1432 | 9.47 |  | 19 | 1.11 |  |  |
| **rs1229984** |  |  |  |  |  |  | 0.0086 |
| **TT** | 8238 | 54.49 |  | 921 | 53.61 |  |  |
| **TC** | 5915 | 39.12 |  | 653 | 38.01 |  |  |
| **CC** | 966 | 6.39 |  | 144 | 8.38 |  |  |
| ***Hemorrhagic stroke*** | | | | | | | |
| **rs671** |  |  |  |  |  |  | 0.0658 |
| **GG** | 23 | 40.35 |  | 10 | 76.92 |  |  |
| **GA** | 28 | 49.12 |  | 3 | 23.08 |  |  |
| **AA** | 6 | 10.53 |  | 0 | 0 |  |  |
| **rs1229984** |  |  |  |  |  |  | 0.0188 |
| **TT** | 36 | 63.16 |  | 3 | 23.08 |  |  |
| **TC** | 16 | 28.07 |  | 9 | 69.23 |  |  |
| **CC** | 5 | 8.77 |  | 1 | 7.69 |  |  |
| ***Ischemic stroke*** | | | | | | | |
| **rs671** |  |  |  |  |  |  | <.0001 |
| **GG** | 382 | 48.79 |  | 76 | 71.03 |  |  |
| **GA** | 334 | 42.66 |  | 29 | 27.1 |  |  |
| **AA** | 67 | 8.55 |  | 2 | 1.87 |  |  |
| **rs1229984** |  |  |  |  |  |  | 0.3148 |
| **TT** | 422 | 53.90 |  | 55 | 51.40 |  |  |
| **TC** | 315 | 40.23 |  | 49 | 45.79 |  |  |
| **CC** | 46 | 5.87 |  | 3 | 2.80 |  |  |
